# Oral hygiene is independently associated with early stroke-associated pneumonia in hospitalized patients with ischemic stroke: An observational study

**DOI:** 10.1097/MD.0000000000041758

**Published:** 2025-03-07

**Authors:** Xiaohua Yang, Yingqiong Lu, Huijuan Chen, Juan Wei, Juan He, Juan Zhang, Tingting Hu, Qing Wang, Xi Tao

**Affiliations:** a Department of Neurology, Zhujiang Hospital, Southern Medical University, Guangzhou, Guangdong Province, China; b School of Rehabilitation Sciences, Southern Medical University, Guangzhou, Guangdong Province, China; c Department of Nursing, Hunan Provincial People’s Hospital, Hunan Normal University, Changsha, Hunan Province, China; d Department of Neurological Rehabilitation, Hunan Provincial People’s Hospital, Hunan Normal University, Changsha, Hunan Province, China; e Clinical Research Center for Cerebrovascular Disease Rehabilitation in Hunan Province, Changsha, Hunan Province, China; f Clinical Medicine Research Center for Respiratory Rehabilitation in Hunan Province, Changsha, Hunan Province, China.

**Keywords:** evaluation, ischemic stroke, nursing, oral hygiene, stroke-associated pneumonia

## Abstract

Stroke-associated pneumonia (SAP) is a major stroke complication. Oral microorganisms are important contributors to SAP. Here, we aimed to investigate whether oral hygiene is associated with early SAP (<72 h of stroke onset) in patients with ischemic stroke. We performed an observational study of 331 patients with acute ischemic stroke from 2 medical centers. A series of assessments were performed to evaluate the neurological status, Beck Oral Assessment Scale (BOAS), and habits of oral hygiene. Univariate and binary logistic regression analyses were conducted to identify the risk factors for early SAP. Potentially relevant factors for oral hygiene in patients with early SAP and general ischemic stroke were also analyzed. Older age, higher prevalence of coronary heart disease, dysphagia and feeding with stomach tube, shorter course of disease as well as severe neurological impairments (such as National Institutes of Health Stroke Scale and Mini-Mental State Examination) were occurred in patients with early SAP (vs non-SAP, all *P* < .05). After adjusting for confounders, the analysis showed that BOAS score (odds ratio [OR] = 1.972, 95% confidence interval [CI] [1.479, 2.630], *P* < .001) and National Institutes of Health Stroke Scale (OR = 1.322, 95% CI [1.211, 1.443], *P* < .001) were independent risk factors for early SAP (OR = 1.678, *P* = .001). The correlation between BOAS scores and potential variables showed sex-dependent differences in patients with early SAP (all *P* < .05). The severity of neurologic impairment, age, and number of dental caries may be factors that influence abnormal BOAS scores (vs normal BOAS scores, all *P* < .05). Abnormal oral hygiene was an independently associated factor in the assessment of early SAP. Emphasis on the relevant influences on oral health may be a nursing strategy for reducing the occurrence of SAP.

## 1. Introduction

Stroke-associated pneumonia (SAP) is a major complication of stroke that significantly increases morbidity and mortality.^[1–3]^ A consensus that treatment for early SAP (<72 h after stroke onset) should cover community-acquired pneumonia organisms was reached by the PISCES group.^[4]^ However, the pathogenesis of SAP remains complex. In addition to immunosuppression induced by stroke,^[4,5]^ dysphagia, poor respiratory muscle strength, decreased pulmonary function, and respiratory center dysfunction after stroke also play important roles.^[4–7]^ Among them, oral hygiene, which may carry a large number of pathogenic microorganisms, is often overlooked.

Several lines of recent evidence indicate that oral hygiene may be an important contributor to lung infections.^[1,7]^ Patients with stroke often have difficulties maintaining oral health for a variety of reasons, such as cognitive impairment and oral functional disabilities.^[5–8]^ Therefore, a tremendous increase in oral pathogens not only induces the incidence of oral infections but also increases the risk of lower respiratory tract infections. Previous studies have demonstrated that oral hygiene care can lower the risk of pneumonia in both nonventilated and ventilated patients.^[9–13]^ Furthermore, another study suggested that systematic oral hygiene care can decrease the risk of hospital-acquired pneumonia (HAP) in patients with acute subacute stroke.^[1,7]^ These results indicate that maintaining oral health in patients with stroke may help to reduce the risk of SAP and improve the prognosis.^[8]^ To our knowledge, the relationship between oral hygiene and early SAP in patients with ischemic stroke is limited. Based on the strong demand for nursing interventions, this study analyzed the factors that may be of interest in the early evaluation of SAP.

## 2. Methods

### 2.1. Patients information

From May 2019 to April 2020, we conducted a retrospective clinical data analysis at Zhujiang Hospital of Southern Medical University and Hunan Provincial People’s Hospital, involving hospitalized patients with early ischemic stroke. No interventions were applied.

The inclusion criteria were as follows: (1) all patients were conscious; (2) all patients were admitted for new-onset cerebrovascular events and confirmed by brain computed tomography (CT, 64-row multidetector) or magnetic resonance imaging (1.5 T); and (3) all early SAP was diagnosed by a physician according to the consensus of PISCES criteria^[14]^ and confirmed by chest CT (<72 h of stroke onset).^[15]^

The exclusion criteria were as follows: (1) hemorrhagic stroke; (2) severe aphasia; (3) noncooperation with Mini-Mental State Examination (MMSE) evaluation; (4) tracheotomy or oral tracheal intubation; (5) primary oral diseases, such as tongue or oral cancer; (6) severe liver (alanine aminotransferase >200 U/L) or kidney (plasma creatinine >186 μmol/L) dysfunction; (7) toothlessness; (8) noncooperation with Beck Oral Assessment Scale (BOAS) inspection; and (9) pneumonia before stroke onset.

The sample size was estimated using a rule of thumb, approximately 10 to 20 times the number of variables.^[16,17]^ We selected the higher value, resulting in an estimated 320 patients. Relevant information and assessment forms were digitized into an electronic questionnaire via the Wenjuanxing app for ease of data collection on mobile devices. The collected data were then imported into Excel and SPSS for analysis.

**Figure 1.**
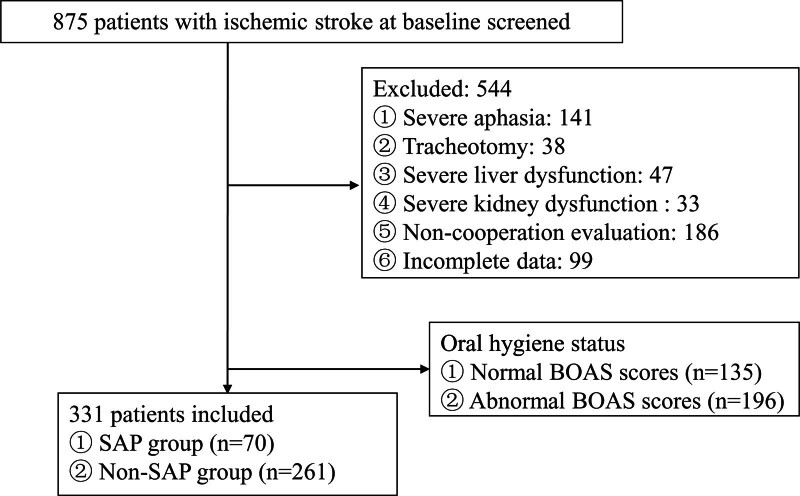
Flow diagram of study selection process. BOAS = Beck Oral Assessment Scale, SAP = stroke-associated pneumonia.

Following screening, 875 ischemic stroke patients were assessed. Of these, 544 were excluded: 141 with severe aphasia, 38 with tracheostomy, 47 with severe liver dysfunction, 33 with severe renal dysfunction, 186 unable to cooperate with assessments, and 99 with incomplete data. A total of 331 patients were included in the study (Fig. 1).

The outcome variable was SAP. Patients were classified into SAP and non-SAP groups based on the presence or absence of early SAP. To further explore variable associations, ischemic stroke patients were grouped into normal and abnormal oral hygiene categories according to the BOAS scoring system.

### 2.2. Data collection

Basic demographic information, including sex, age, educational background, disease course, number of stroke events, vascular risk factors (hypertension, diabetes, coronary heart disease, smoking, and body mass index [BMI]), and gastrointestinal diseases (at the time of investigation), was collected from all patients. Neurological function assessments included the National Institutes of Health Stroke Scale (NIHSS), used to assess neurological deficits; the Barthel index (BI), used to assess the activities of daily living; the MMSE, used to assess cognitive function; and the water swallowing test (WST) scale, used to screen swallowing function in patients with ischemic stroke.^[18]^

### 2.3. Ethical approval

This observational study was approved by the Ethics Committees of Zhujiang Hospital of Southern Medical University and Hunan Provincial People’s Hospital of Hunan Normal University (Human Ethics Number: 2020-KY-015-01) and was conducted according to the principles outlined in the revised Declaration of Helsinki of 1975 and the National Institutes of Health Human Subjects Policies and Guidelines released in 1999. All the participants signed an informed consent form for the questionnaire survey.

### 2.4. Definition of terms

SAP refers to radiologically detectable pulmonary inflammation within 7 days in non-mechanically ventilated stroke patients, potentially linked to secondary immune dysfunction following central nervous system injury.^[14]^ In this study, early SAP was defined as SAP diagnosed within 3 days of stroke onset.^[4]^

BOAS was used to assess the oral cleanliness of all patients.^[19,20]^ The BOAS scale consists of 5 items: lips, gums and oral mucosa, teeth, tongue, and saliva. Each item is scored on a 4-point scale ranging from 1 to 4 points. A total BOAS score of 5 is considered normal, 6 to 10 as mild dysfunction, 11 to 15 as moderate dysfunction, and 16 to 20 as severe dysfunction.

The NIHSS is widely used to evaluate the severity of neurological deficits in stroke patients. It includes 11 assessment items: consciousness, gaze, visual fields, facial palsy, upper and lower limb motor function, ataxia, sensation, language, dysarthria, and neglect.^[21]^ A higher score reflects more severe neurological impairment.

The MMSE is widely used for cognitive screening, comprising 30 subtests that assess orientation, memory, calculation, naming, reading comprehension, and other functions.^[22]^ It offers good sensitivity and specificity and is clinically convenient. The total score is 30, with lower scores indicating greater cognitive impairment.

The BI is used to assess daily living activities, including bowel and bladder control, grooming, toileting, feeding, transferring, mobility, dressing, stair climbing, and bathing.^[23]^ The total score is 100, with lower scores indicating poorer self-care ability.

Based on the knowledge of the nursing team members and previous literature,^[24,25]^ we used a scale with self-variable indicators to investigate the recent (<3 days) oral hygiene habits, care styles, and objective conditions related to the oral cavities of ischemic stroke patients. The scale contains 10 items: the number of dental caries, oral cleaners, frequency of cleaning, attention to oral hygiene, number of cleansing sites (lips, tongue, gums, oral mucosa, and teeth), whether active dentures are used, mouthwash, dental floss, toothbrushes, and the stomach tube. The BOAS and scales with self-variable indicators were evaluated by 2 specialist nurses who were not blinded, and other assessments were performed by 2 non-blinded physicians on the first day after admission. Systematic training and guidance were provided prior to the investigation to reduce bias in understanding the assessment during the clinical data survey.

### 2.5. Statistical analysis

All data were analyzed using SPSS 17.0 (IBM Corporation, Armonk, NY). The exploratory analysis method (Kolmogorov–Smirnov) was used to test whether the measurement data of each group followed a normal distribution. The *t* test was used to compare 2 normally distributed, homoscedastic, and independent samples. The Mann–Whitney *U* method was used to compare 2 non-normally distributed independent samples and grade data. The chi-square test was used to compare the percentage-based data between the 2 groups. The Spearman correlation coefficient was used to express the correlation between 2 variables (because there may be differences in lifestyle habits between women and men, patients were classified into 2 groups for further correlation analysis). The Box–Tidwell method was used to test whether there was a linear relationship between a continuous independent variable and the dependent variable after logit conversion. Tolerance and variance inflation factors were used to exclude collinearity between the independent variables. Univariate analysis was performed on these data, and variables with *P* < .2, indicating potential risk factors were included in the multivariable logistic stepwise regression analysis. Variables were retained if the corresponding *P* values ≤ .05 and rejected if *P* > .1. The Hosmer–Lemeshow test was used to judge the goodness of fit of the regression model. The model was developed to evaluate the risk factors for early SAP in patients with ischemic stroke. Finally, the receiver operating characteristic (ROC) curve was determined to evaluate the probability value of the model. Data analysts were not involved in the scale evaluation and their main work was data analysis.

## 3. Results

### 3.1. Patient characteristics

A total of 875 patients diagnosed with ischemic stroke were screened, 544 of whom were excluded according to the exclusion criteria, and 331 patients were finally enrolled for further analysis. The mean age (standard deviation) of the enrolled patients was 64.38 (11.65), and 67.07% were male (Table 1). Seventy early SAP were identified and collectively constituted the SAP group, and 261 patients with ischemic stroke without SAP were selected as the non-SAP group (Fig. 1).

**Table 1:** Comparison of basic demographic data between SAP and non-SAP groups.

Variables	SAP group (n = 70)	Non-SAP group (n = 261)	*χ* ^2^ */Z*	*P*
Sex (male)	53 (75.71%)	169 (64.75%)	3.004*	.083
Hypertension	54 (77.14%)	173 (66.28%)	3.021*	.082
Diabetes mellitus	24 (34.29%)	75 (28.74%)	0.811*	.368
Coronary heart disease	14 (24.29%)	16 (6.13%)	12.883*	**<.001**
Smoking	32 (45.71%)	104 (39.84%)	0.785*	.376
Gastrointestinal diseases	4 (5.71%)	29 (11.11%)	1.791*	.181
Stomach tube	14 (20.00%)	5 (1.91%)	25.969†	**<.001**
WST				
Normal	46 (65.71%)	223 (85.44%)	25.238†	**<.001**
Doubtful	9 (12.86%)	31 (11.88%)		
Abnormal	15 (21.43%)	7 (2.68%)		
Age, years§	68.50 (18.25)	64.00 (14.00)	‐2.120‡	**.034**
Course of disease, hours§	4.50 (3.00)	5.00 (6.00)	‐3.022‡	**.003**
Num. of stroke§	1.00 (0.00)	1.00 (0.00)	‐0.217‡	.828
BMI§	23.33 (4.02)	23.60 (4.48)	‐1.742‡	.082
NIHSS§	6.00 (7.25)	3.00 (2.00)	‐9.940‡	**<.001**
BI§	62.50 (51.25)	95.00 (20.00)	‐4.857‡	**<.001**
MMSE§	20.00 (12.00)	26.00 (6.00)	‐7.279‡	**<.001**
BOAS score§	7.00 (2.25)	6.00 (1.00)	‐6.228‡	**<.001**

Bold values indicate statistical significance <.05.

BI = Barthel index, MMSE = Mini-Mental State Examination, BMI = body mass index, BOAS = Beck Oral Assessment, NIHSS = National Institutes of Health Stroke Scale, Num. = number, SAP = stroke-associated pneumonia, SD = standard deviation, WST = water swallowing test.

* Pearson *χ*^2^ test.

† Likelihood ratio test.

‡ Mann–Whitney *U* test.

§ Shown as median (IQR).

### 3.2. Comparison of various factors between SAP and non-SAP groups

Age (*Z* = ‐2.120, *P* = .034), BOAS (*Z* = ‐6.228, *P* < .001), and NIHSS scores (*Z* = ‐9.940, *P* < .001) were higher in the SAP group than in the non-SAP group, whereas the course of disease (*Z* = ‐3.022, *P* = .003), BI (*Z* = ‐4.857, *P* < .001), and MMSE scores (*Z* = ‐7.279, *P* < .001) were lower in the non-SAP group. The percentages of abnormal WST (*χ*^2^ = 25.238, *P* < .001) and indwelling stomach tube (*χ*^2^ = 25.969, *P* < .001) were higher in the SAP group than in the non-SAP group. In addition, the percentage of patients with coronary heart disease was higher in the SAP group than in the non-SAP group (*χ*^2^ = 12.883, *P* < .001). There were no significant differences in other variables (Table 1).

### 3.3. Correlations analyses between BOAS scores and clinical variables in early SAP patients

Spearman’s correlation analysis was performed to evaluate the relationship between BOAS scores and clinical variables in patients with early SAP. We found that the BOAS score (total population, n = 70) was positively correlated with the number of dental caries (*r*_s_ = 0.307, *P* = .010) (Table 2) and negatively correlated with oral hygiene awareness (*r*_s_ = −0.357, *P* = .002) (Table 2).

**Table 2:** Correlations analyses between BOAS scores and clinical variables in early SAP patients.

Variables	BOAS score (total, n = 70)		BOAS score (male, n = 53)		BOAS score (female, n = 17)	
	*r*_s_	*P*	*r*_s_	*P*	*r*_s_	*P*
Age, years	‐0.002	0.984	‐0.092	0.513	0.248	0.338
Course of disease, hours	‐0.066	0.590	‐0.097	0.491	0.037	0.888
Num. of stroke	‐0.113	0.353	0.038	0.787	‐0.367	0.147
Num. of dental caries	0.307	**0.010**	0.250	0.070	0.473	0.055
Frequency of cleaning	0.065	0.595	0.000	0.996	0.230	0.374
Oral hygiene awareness	‐0.357	**0.002**	‐0.230	0.097	‐0.685	**0.002**
Num. of cleansing sites	‐0.124	0.305	‐0.144	0.304	‐0.091	0.729
BMI	‐0.222	0.065	‐0.068	0.628	‐0.654	**0.004**
NIHSS	0.220	0.067	0.071	0.615	0.700	**0.002**
MMSE	‐0.120	0.322	0.050	0.721	‐0.629	**0.007**
BI	‐0.119	0.325	0.017	0.905	‐0.498	**0.042**

BI = Barthel index, BMI = body mass index, BOAS = Beck Oral Assessment Scale, MMSE = Mini-Mental State Examination, NIHSS = National Institutes of Health Stroke Scale, Num. = number.

In addition, the patients were classified into 2 groups for further analysis. In the female group (n = 17), the BOAS score was positively correlated with the NIHSS score (*r*_s_ = 0.700, *P* = .002) (Table 2), whereas strong negative correlations were found with BMI (*r*_s_ = −0.654, *P* = .004), BI (*r*_s_ = −0.498, *P* = .042), MMSE score (*r*_s_ = −0.629, *P* = .007), and oral hygiene awareness (*r*_s_ = −0.685, *P* = .002) (Table 2). However, in the male group (n = 53), no definite correlation was found with the BOAS score (Table 2).

### 3.4. Binary logistic regression analysis in early SAP patients

Variables with a univariate analysis (*P*-value < .2) shown in Table 1 were included in the risk factor analysis. Logistic regression analysis was performed using SAP as the dependent variable. Ultimately, 13 independent variables were included in the model. The results showed that BOAS score (odd ratios [OR] = 1.972, 95% confidence interval [CI] [1.479, 2.630], *P* < .001) was the primary risk factor for early SAP in hospitalized patients with ischemic stroke, whereas NIHSS score (OR = 1.322, 95% CI [1.211, 1.443], *P* < .001) was the secondary risk factor (Table 3).

**Table 3:** Logistic regression analysis with early SAP as the dependent variable.

Risk factors	*B*	SE	*P*	OR (95% CI)
BOAS score	0.679	0.147	**<.001**	1.972 (1.479, 2.630)
NIHSS score	0.279	0.045	**<.001**	1.322 (1.211, 1.443)

B = unstandardized coefficients, BOAS = Beck Oral Assessment Scale, NIHSS = National Institutes of Health Stroke Scale, OR = odds ratio, SAP = stroke-associated pneumonia, SE = standard error.

### 3.5. ROC curves for risk factors in the evaluation of early SAP

**Figure 2.**
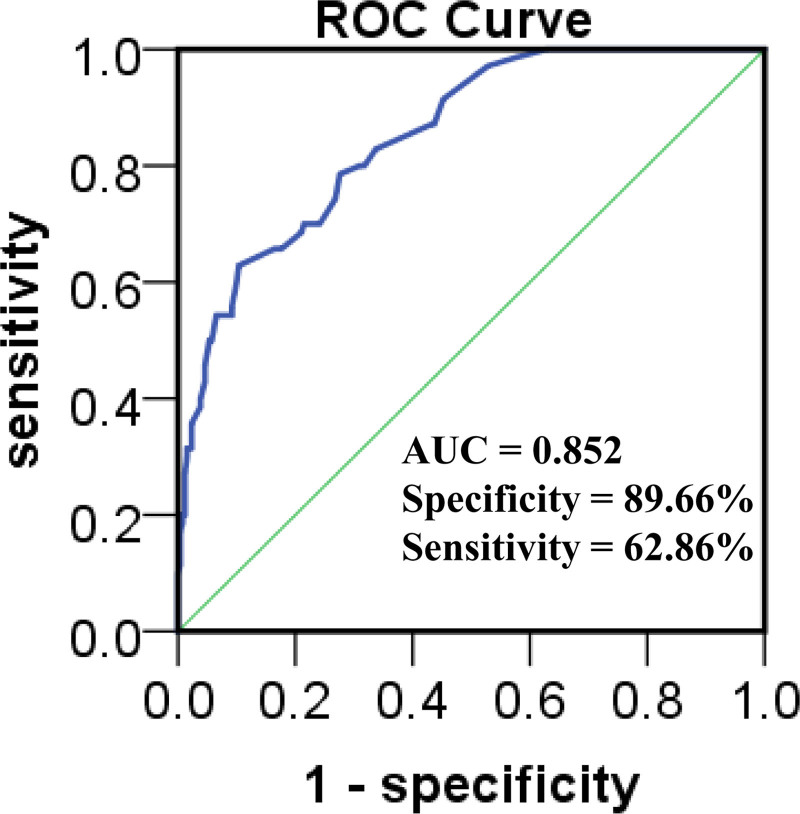
ROC curve for combined BOAS with NIHSS scoring to evaluate early SAP in patients with ischemic stroke. BOAS = Beck Oral Assessment Scale, NIHSS = National Institutes of Health Stroke Scale, ROC = receiver operating characteristic, SAP = stroke-associated pneumonia.

ROC curves were constructed to explore whether the BOAS and NIHSS scores could evaluate early SAP in hospitalized patients with ischemic stroke. The area under the ROC curve of the combination of BOAS and NIHSS was 0.852, with a standard error of 0.024 (*P* < .001, 95% CI [0.805, 0.898]). The cutoff was 0.277, with a specificity of 89.66%, sensitivity of 62.86%, and accuracy of 81.27% (Fig. 2). The Hosmer–Lemeshow test showed *χ*^2^ = 5.231, df = 8, and *P* = .411.

### 3.6. Comparisons of baseline data in oral hygiene between normal and abnormal BOAS scores (groups)

To further analyze the potential factors influencing oral health disparities, the patients were divided into a normal BOAS group (n = 135) and an abnormal BOAS group (n = 196) (Table 4). In the abnormal BOAS group, 193 patients had mild dysfunction and 3 had moderate dysfunction.

**Table 4:** Comparison of related data between SE normal and abnormal BOAS groups.

Variables	Normal BOAS groups (n = 135)	Abnormal BOAS groups (n = 196)	*χ* ^2^ */Z*	*P*
Age, years§	63.00 (21.00)	66.50 (14.00)	‐3.739‡	**<.001**
Sex (male)	88 (65.19%)	134 (68.37%)	0.367*	.545
Hypertension	97 (71.85%)	130 (66.33%)	1.133*	.287
Diabetes mellitus	34 (25.19%)	65 (33.16%)	2.427*	.119
Coronary heart disease	11 (8.15%)	19 (9.69%)	0.232*	.630
Smoking	56 (41.48%)	80 (40.82%)	0.015*	.904
BMI§	23.60 (4.77)	23.39 (4.09)	0.846‡	.398
Gastrointestinal diseases	11 (8.15%)	21 (10.71%)	0.603*	.438
Stomach tube	3 (2.22%)	16 (8.16%)	5.215*	**.022**
Course of disease, hours§	5.00 (6.00)	5.00 (4.00)	‐0.885†	.376
Num. of stroke§	1.00 (0.00)	1.00 (0.00)	‐1.058†	.290
NIHSS§	2.00 (2.00)	3.00 (3.00)	‐3.951†	**<.001**
BI§	100.00 (20.00)	90.00 (40.00)	‐4.189†	**<.001**
MMSE§	26.00 (7.00)	25.00 (7.00)	‐2.497†	**.013**
Active dentures	19 (14.07%)	24 (12.24%)	0.237*	.627
Oral cleaners	126 (93.33%)	172 (87.76%)	2.771*	.096
Toothbrush	130 (96.30%)	180 (91.84%)	2.676*	.102
Num. of dental caries§	0.00 (2.00)	1.00 (2.00)	‐3.615†	**<.001**
Frequency of cleaning§	2.00 (1.00)	2.00 (1.00)	‐0.471†	.638
Num. of cleaning sites§	1.00 (1.00)	1.00 (1.00)	‐1.316†	.188
Education				
Illiteracy	24 (17.78%)	23 (11.73%)	‐0.491†	.623
Primary	31 (22.96%)	57 (29.08%)		
Secondary	32 (23.70%)	62 (31.63%)		
High	30 (22.22%)	38 (19.39%)		
University or above	18 (13.33%)	16 (8.16%)		
WST				
Normal	122 (90.37%)	147 (75.00%)	‐3.586†	**<.001**
Doubtful	10 (7.41%)	30 (15.31%)		
Abnormal	3 (2.22%)	19 (9.69%)		
Attention of oral hygiene				
Unimportant	9 (6.67%)	18 (9.18%)	‐0.166†	.868
General	109 (80.74%)	148 (75.51%)		
Crucial	17 (12.59%)	23 (11.73%)		
All-important	0 (0.00%)	7 (3.57%)		
SAP	14 (10.37%)	56 (28.56%)	15.881*	**<.001**

BI = Barthel index, BMI = body mass index, BOAS = Beck Oral Assessment Scale, MMSE = Mini-Mental State Examination, NIHSS = National Institutes of Health Stroke Scale, Num. = number, SAP = stroke-associated pneumonia, SD = standard deviation, WST = water swallowing test.

* Pearson *χ*^2^ test.

† Mann–Whitney *U* test.

‡ Student’s *t* test.

§ Shown as median (IQR).

In this study, patients with abnormal oral hygiene were more likely to present with swallowing dysfunction identified by the WST (*χ*^2^ = ‐3.586, *P* < .001) and use of an indwelling stomach tube (*χ*^2^ = 5.215, *P* = .022) than patients in the normal group. The age (*t* = ‐3.739, *P* < .001) and number of dental caries (*Z* = ‐3.615, *P *< .001) in the abnormal BOAS group were higher than those in the normal group. The BI (*Z* = ‐4.189, *P* < .001) and MMSE scores (*Z* = ‐2.497, *P* = .013) were lower, and the NIHSS score (*Z* = ‐3.951, *P* < .001) was higher in the abnormal than in the normal BOAS group. Moreover, the incidence of SAP in the abnormal BOAS group was higher than that in the normal BOAS group (*χ*^2^ = 15.881, *P* < .001). Although the proportion of patients who could independently perform mouth cleaning, the use of a toothbrush, and the number of cleaning sites in the abnormal BOAS group tended to be lower than those in the normal group, the differences were not significant (Table 4).

## 4. Discussion

Our study revealed the following 3 findings. First, the BOAS and NIHSS scores were independent risk factors for early SAP. Second, the correlation between BOAS scores and various potential risk factors showed sex-dependent differences in the patients with early SAP. Third, the severity of neurological impairment, age, and dental caries may be factors that influence inadequate oral hygiene. To our knowledge, this is the first investigation and assessment model of oral hygiene associated with early SAP, which may be helpful in guiding clinical nursing work.

Previous studies have shown that HAP is closely related to oral health.^[26,27]^ Any inflammation or damage to the oral mucosa could promote bacterial adhesion, colonization, reproduction, and infection.^[28,29]^ Migration of this infection through the upper respiratory tract to the lower respiratory tract is common in clinical practice.^[6]^ Therefore, maintaining the integrity of the oral mucosa through daily care is important to prevent secondary nosocomial infections.^[29]^ However, oral care is not limited to the oral mucosa; the gums, teeth, lips, tongue, and even salivation are included in daily clinical nursing work. The BOAS is a scale containing the 5 abovementioned items that should be addressed in oral care.^[19]^ The scale is not only used for the evaluation of nursing care but also to guide nursing intervention for critically ill patients.^[19,20,30]^

Among critically ill patients, stroke patients are susceptible to HAP not only at the early stage but also in the recovery period, and the incidence of acquired pneumonia among stroke patients is significantly higher than that of the general patient population.^[6,31]^ However, over the past decade, SAP has become widely recognized as a new type of pneumonia for its mechanisms and harmful effects.^[1,2,7,32,33]^ The causes of this complication may be related to the stroke-induced immunosuppression, weakening of the pharyngeal reflex, the loss of proprioception or superficial sensation of the oral mucosa, and a weakening of the ability of the lips and tongue to push food into the pharynx after brain injury.^[5,7,8,14,34]^ Therefore, screening for risk factors for SAP and establishment of an assessment model can help to reduce the occurrence of SAP from a nursing perspective.^[7]^

In this study, we analyzed 70 patients with early SAP independently and compared them with non-SAP patients. The results showed significant differences in the 9 variables between SAP and non-SAP patients. Higher age and prevalence of coronary heart disease, as well as severe neurological impairment (NIHSS, MMSE, etc), are important features of early SAP. The shorter course of the disease in patients with SAP may be related to their earlier and more severe presentations. Interestingly, the SAP group had significantly higher rates of dysphagia and feeding with the stomach tube, especially with higher BOAS scores, than the non-SAP group. Therefore, poor oral hygiene is a risk factor of SAP. This point has not been raised in the literature, and we performed further analysis.

In general, abnormal oral hygiene is closely related to personal hygiene habits. However, this situation may change in the presence of certain diseases, such as nutritional or exercise-induced immune suppression, long-term use of antibiotics, dehydration, etc.^[35–38]^ Little has been reported regarding whether oral hygiene affect early SAP.^[36]^ This study found that abnormal oral hygiene may be the leading risk factor for early SAP, whereas neurologic deficits were secondary in patients with ischemic stroke, although the exact reason is not clear. The latter finding is consistent with previous reports.^[33]^ The results suggest that abnormal oral hygiene has a higher risk coefficient than swallowing disorders, which is different from previous studies.^[5,33]^

In addition, our results showed that BOAS score was only positively related to the number of dental caries and negatively related to oral health concerns. A causal relationship exists between oral cleanliness and caries, which may worsen with age. It is important for us and for guidance practices or nurses to pay greater attention to the internal relationship between caries and oral health after ischemic stroke. Interestingly, this study showed that the correlations between BOAS scores and clinical variables were sex dependent. In female patients, BMI, instead of the number of dental caries, was closely related to the BOAS score. Furthermore, the severity of neurological impairment (NIHSS, MMSE, etc) was strongly correlated with the BOAS score. Studies have shown that cognitive impairment and low individual activity levels may be potential risk factors for oral hygiene abnormalities.^[39–41]^ This is consistent with the results of previous studies. Female patients had more relevant indicators, whereas male patients had fewer. The specific reason for this difference is unknown.

Dental caries are difficult to change in the short term and are an important intraoral factor that influences oral health. Caries are recognized as 1 of the 3 major human diseases, including cancer and cardiovascular diseases.^[42,43]^ Its etiology is complex and may be related to bacteria, the oral microenvironment and the time of action.^[44]^ Carbohydrates are the main source of food for the Chinese people. A small amount of starch is easily deposited on the tooth surface after a meal and then forms a mixture with the trace protein in the saliva. With a suitable temperature and sufficient time, bacteria, parasites, and viruses can easily reproduce, and visible plaques and unpleasant oral odors are quickly generated.^[43,45]^ From the perspective of pathophysiology, caries is the result of long-term adhesion of plaque to teeth and destruction of organic matter.^[44,45]^ Patients with dental caries are more likely to have food residues in the cavities after meals, which makes it difficult to completely remove plaque. Therefore, caries are likely to be one of the causes of poor oral health. Comparatively, this situation is worse in patients with stroke.^[24]^

In addition to caries, age, use of indwelling gastric tubes, and WST, NIHSS, and MMSE scores were anatomically or functionally closely related to abnormal oral cleanliness in stroke patients. These results indicate that: (1) the risk of oral hygiene abnormalities increases with age; (2) objective abnormalities of the internal anatomy or pathological structure of the oropharynx and/or respiratory tract may be an important factor in abnormal oral hygiene; and (3) cognitive function strengthening and activities of daily livings rehabilitation may help improve oral hygiene in patients with acute ischemic stroke.

Some limitations of this study should be noted: (1) a relatively small sample size of patients with early SAP was included in the present study; (2) to reduce the SAP diagnosis bias, case collection time should have been extended to the 7th day of the disease course; (3) patients with severe aphasia or tracheotomy were not included; and (4) biochemical indicators, such as inflammatory factors, were not included as potential risk factors. Therefore, an increase in the sample size and observational markers included can help to further analyze the role of oral hygiene in patients with early SAP.

## 5. Conclusion

Abnormal oral hygiene was an independently associated factor in the assessment of early SAP. Sex-dependent differences in oral hygiene among patients with SAP may exist. Emphasis on the relevant effects on oral health may be a strategy to reduce the occurrence of SAP.

## Author contributions

**Conceptualization:** Xiaohua Yang, Huijuan Chen, Qing Wang, Xi Tao.

**Data curation:** Xiaohua Yang, Huijuan Chen, Juan Wei, Juan Zhang.

**Formal analysis:** Juan He.

**Funding acquisition:** Xiaohua Yang, Juan He, Xi Tao.

**Investigation:** Xiaohua Yang, Huijuan Chen, Juan Wei, Juan He, Juan Zhang, Tingting Hu.

**Methodology:** Juan He, Tingting Hu, Xi Tao.

**Writing – original draft:** Xiaohua Yang, Yingqiong Lu, Qing Wang, Xi Tao.

**Writing – review & editing:** Yingqiong Lu, Qing Wang, Xi Tao.
